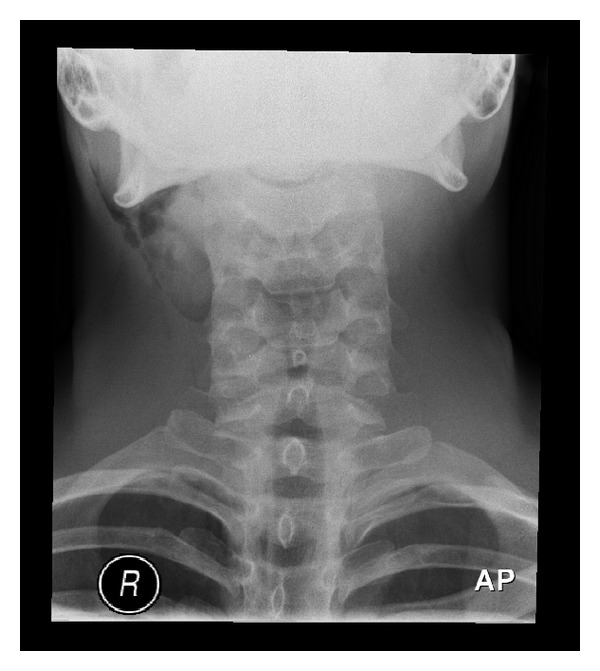# Cervicofacial Surgical Emphysema following Tonsillectomy

**DOI:** 10.1155/2014/746152

**Published:** 2014-05-08

**Authors:** Samir Yelnoorkar, Wolfgang Issing

**Affiliations:** ENT Department, Freeman Hospital, Freeman Road, High Heaton, Newcastle upon Tyne, NE7 7DN, UK

## Abstract

We report the case of a patient who developed cervicofacial subcutaneous emphysema following a routine tonsillectomy. An 18-year-old male with swallowing difficulties underwent a tonsillectomy and developed swelling of the right side of his neck and face 36 hours after surgery. A neck X-ray revealed subcutaneous emphysema. Unlike similar previously published cases, there were no postoperative issues of coughing, straining, or use of positive pressure ventilation. The complication also occurred after a considerable length of time. Further complications may include pneumothorax and pneumomediastinum and these should be excluded.

## 1. Introduction

Tonsillectomy is a common ear, nose, and throat procedure with hemorrhage being the most significant risk. Surgical emphysema is an infrequent [1] but potentially serious complication of tonsillectomy [2, 3]. Although most cases are self-limiting and can be managed conservatively, early recognition and prompt treatment can prevent further complications [3]. We report the case of an 18-year-old male who developed cervicofacial subcutaneous emphysema. The case was unusual in that the patient developed this complication 36 hours after tonsillectomy. The likely mechanism in this case will be discussed.

## 2. Case Presentation

An 18-year-old male with a long history of swallowing difficulties was admitted for tonsillectomy. He described dysphagia that was worse for solids than liquids and felt that food was sticking at the back of his throat. Swallowing solids was made possible when he extended his neck. Past medical history included asthma that was well controlled. There was no history of recurrent tonsillitis or obstructive sleep apnoea. Examination revealed grossly enlarged tonsils that were almost meeting at the midline. Barium swallow revealed normal flow through the pharynx and oesophagus.

Tonsillectomy was performed under general anaesthesia with endotracheal intubation. Intubation was nontraumatic and uneventful. The tonsils were dissected using bipolar diathermy and haemostasis was achieved with bipolar electrocautery. The operation proceeded uneventfully with no intraoperative or immediate postoperative complications. There were no anaesthetic difficulties and the patient recovered within 15 minutes. Late postoperative progress was satisfactory with no episodes of coughing, straining, or vomiting. The patient was discharged the following day with minimal throat discomfort and good oral intake.

He was subsequently readmitted 36 hours after surgery with generalised swelling of his right face and neck. Physical examination revealed marked crepitus on palpation consistent with cervicofacial subcutaneous emphysema. There was no evidence of cardiorespiratory compromise. Normal postoperative features were noted in the oropharynx, with no visible muscle dehiscence or mucosal trauma. Soft tissue neck radiographs revealed that air was confined to the right neck and parotid region (Figure 1). A chest radiograph was normal with no evidence of pneumothorax or pneumomediastinum.

The patient was commenced on broad-spectrum prophylactic antibiotics and admitted for observation. The subcutaneous emphysema began to resolve and the patient made a full recovery. He was discharged on the third postoperative day.

## 3. Discussion

Subcutaneous emphysema is a rare complication of tonsillectomy and has been reported back as far as 1933 [4]. A literature search revealed at least 34 published cases. The degree of emphysema varied and included bilateral cases, pneumomediastinum, pneumothorax, and rarely pneumoperitoneum. In the vast majority of these cases, conservative management was successful. In rare instances, respiratory compromise mandated a period of ventilation by intubation or tracheostomy [3, 5].

The mechanism leading to subcutaneous emphysema has not been conclusively identified. A likely explanation is that deep dissection into the tonsil fossa causes a breach of the superior constrictor muscle and the underlying fascia. This provides a route for air to enter the parapharyngeal space. The process is facilitated by events such as coughing or straining that increase upper airway pressure. Air may then spread into the retropharyngeal space, mediastinum, and even the peritoneum via the diaphragmatic openings. Mediastinitis is a rare but potentially fatal outcome [6]. Other forms of trauma to the upper aerodigestive tract mucosa such as traumatic intubation are also implicated [7]. One reported case describes a man falling with a pipe in his mouth resulting in penetrating trauma to the right anterior tonsillar pillar and subsequent subcutaneous emphysema [8].

In some instances, alveolar rupture may be the initial event. Free air can enter the pulmonary interstitium and dissect along the pulmonary vasculature to reach the mediastinum [9] and then track into the head and neck. In our case, there was no pneumomediastinum to support this mechanism and it is more likely that air entered through the right tonsil fossa.

Cervicofacial swelling with crepitus following tonsillectomy should raise suspicion of subcutaneous emphysema. A plain neck radiograph will demonstrate the finding. Pneumothorax and pneumomediastinum should be excluded with physical examination and a chest radiograph. Regular cardiorespiratory assessment should be undertaken, particularly observing the signs of pneumothorax, tracheal compression, and reduced cardiac output. Treatment aims to prevent further air tracking within tissue planes by avoiding actions that increase upper airway pressure such as coughing or straining. Commencement of broad-spectrum antibiotics may prevent development of life threatening mediastinitis.

Subcutaneous emphysema is a rare complication of tonsillectomy that tends to arise intraoperatively or during the immediate postoperative period [10]. This case is unusual in that subcutaneous emphysema developed over 24 hours after tonsillectomy.

## Figures and Tables

**Figure 1:**